# Background phase correction in phase contrast velocity encoded CMR reduces gender differences and improves the accuracy and precision of Qp/Qs

**DOI:** 10.1186/1532-429X-17-S1-P65

**Published:** 2015-02-03

**Authors:** Jannike Nickander, Magnus Lundin, Jonas Jenner, Eva Maret, Peder Sörensson, Andreas Sigfridsson, Martin Ugander

**Affiliations:** 1 Karolinska Institutet, Stockholm, Sweden

## Background

Cardiovascular magnetic resonance (CMR) flow quantification for determining the ratio of flow between the pulmonary and systemic circulation (Qp/Qs) is used to quantify cardiac shunts. However, both the accuracy and precision of Qp/Qs can be influenced by measurement errors in flow quantification. Post-processing methods for stationary tissue background correction have been proposed to improve flow quantification accuracy. We sought to evaluate the changes in accuracy and precision of Qp/Qs following stationary tissue background correction. We hypothesized that stationary background correction would reduce the standard deviation (SD) of mean Qp/Qs compared to uncorrected measurements in clinical patients without cardiac shunts.

## Methods

We enrolled consecutive patients (*n*=120, age 49±16 years, 64% male) referred for clinical CMR at 1.5T (Siemens Aera) in whom the CMR report had no mention of cardiac shunts, malformations in the great vessels or persistent arrhythmias. A clinically available free-breathing non-segmented phase contrast velocity encoded sequence was used for flow imaging in the ascending aorta and pulmonary trunk. Flow quantification was performed both uncorrected and corrected for background phase correction using semi-automated methods with manual adjustments in clinical software (SyngoVia version VA30, Siemens). Exclusion criteria included significant arrhythmia defined as an SD of the mean RR interval during flow acquisition exceeding 10% of RR for either acquisition. Data are given as mean±SD. Means were compared by the paired or unpaired t-test (t) as appropriate, and SDs by the F-test (F).

## Results

94 patients (age 50±16 years, 62% male) had no significant arrhythmia and mean uncorrected Qp/Qs 1.06±0.12 and corrected 1.06±0.09 (t p=0.58, F p=0.007). Males (n=58) had uncorrected Qp/Qs 1.07±0.13 and corrected 1.06±0.09 (t p=0.30, F p=0.006). Females (n=36) had uncorrected Qp/Qs 1.03±0.10 (compared to males t p=0.07, F p=0.12) and corrected 1.07±0.10 (compared to uncorrected t p<0.001, F p=0.67; compared to males t p=0.70, F p=0.72). Compared to females, males had a greater body surface area and body mass index (both p<0.001), but the greatest changes in precision and accuracy for the effect of background correction were obtained for analysis by gender.

## Conclusions

In males, background correction did not change mean Qp/Qs, but improved its precision. In women, background correction increased mean Qp/Qs by four percentage points, with an unchanged precision, yielding a Qp/Qs that did not differ from corrected males. Background correction reduces gender differences and improves the accuracy and precision of Qp/Qs, albeit by different mechanisms in men and women.

## Funding

The research was funded in part by the Swedish Research Council, Swedish Heart and Lung Foundation and the Stockholm County Council. Karolinska Institute has a research and development agreement regarding CMR with Siemens.

